# Impact of social determinants of health on perioperative opioid utilization in patients with lumbar degeneration

**DOI:** 10.1016/j.xnsj.2023.100221

**Published:** 2023-04-19

**Authors:** Aboubacar Wague, Jennifer M. O'Donnell, Khuzaima Rangwalla, Ashraf N. El Naga, David Gendelberg, Sigurd Berven

**Affiliations:** a University of California San Francisco School of Medicine, 505 Parnassus Ave MU 320W, San Francisco, CA 94143, USA; b University of California San Francisco, Department of Orthopaedic Surgery, 505 Parnassus Ave, San Francisco, CA 94143, USA; c Zuckerberg San Francisco General Hospital, 1001 Potrero Ave, San Francisco, CA 94110, USA

**Keywords:** Opioids, Social determinants of health, Lumbar, Degenerative

## Abstract

**Background:**

Social determinants of health (SDOH), have been demonstrated to significantly impact health outcomes in spine patients. There may be interaction between opioid use and these factors in spine surgical patients. We aimed to evaluate the social determinants of health (SDOH) which are associated with perioperative opioid use among lumbar spine patients.

**Methods:**

This retrospective cohort study included patients undergoing spine surgery for lumbar degeneration in 2019. Opioid use was determined based on prescription records from the electronic medical records. Preoperative opioid users (OU) were compared with opioid-naïve patients regarding SDOH including demographics like age and race, and clinical data such as activity and tobacco use. Demographics and surgical data, including age, comorbidities, surgical invasiveness, and other variables were also collected from the records. Multivariate logistic regression was used for analysis of these factors.

**Results:**

Ninety-eight patients were opioid-naïve and 90 used opioids preoperatively. All OU had ≥3 months of use, had more prior spine surgeries (1.07 vs. 0.44, p<.001) and more comorbidities including diabetes, hypertension, and depression (p=.021, 0.043, 0.017). Patients from lower community median income areas, unemployed, or with lower physical capacity (METS<5) were more likely to use opioids preoperatively. Postoperative opioid use was strongly associated with preoperative opioid use, as well as alcohol use, and lower community median income. At one year postoperatively, OU had higher rates of opioid use [72.2% vs. 15.3%, p<.001].

**Conclusions:**

Unemployment, low physical activity level, and lower community median income were associated with preoperative opioid use and longer-term opioid use postoperatively.

## Introduction

Opioid consumption in America is greater than any other country and opioid addiction is a nationwide crisis. In 2018, almost 70,000 Americans died of a drug overdose with two-thirds of these deaths attributed to opioid use [1]. Orthopedics is at the center of the opioid epidemic. Orthopedic surgeons prescribed an estimated 7.7% of opioid prescriptions in the United States in 2009, and accounted for the first prescription of an estimated 8.8% of all opioid-naïve (ON) patients that develop chronic opioid dependence [2,3]. This calls for an assessment of how to better understand and ultimately decrease opioid utilization in orthopedic patients.

Lumbar decompression and fusion surgeries are among the most common spine surgeries. Chronic use of opioids in patients with lumbar degenerative pathology has been correlated to longer hospital stays, increased risk of postoperative complications, and longer opioid use postoperatively [4], [5], [6]. Use of opioids has also been correlated to lower patient-reported outcome measurements [7,8]. To address these issues, it is imperative to identify risk factors that lead to preoperative opioid utilization.

Previous work in other fields have indicated that social conditions such as age, socioeconomic status and smoking status influence the likelihood of chronic opioid use [9,10]. These factors, such as housing status, work status, income, religious belief, insurance status, and marital status, are referred to as social determinants of health (SDoH). The Centers for Disease Control and Prevention (CDC) define SDoH as the “conditions in the places where people live, learn, work, and play that affect a wide range of health and quality of life risks and outcome”. The impact of SDoH on opioid use in orthopedic spine patients has not yet been investigated. This study aims to evaluate the impact of SDoH and health-related behaviors (HRB) on preoperative and postoperative opioid utilization.

### Materials/methods

#### Demographic and opioid utilization

A retrospective cohort study was completed on a consecutive series of patients undergoing elective spine surgery for lumbar degenerative pathology from a single academic institution, from January 2019 to December 2019. Patients who underwent 1- or 2-level lumbar spinal fusion surgery were included if they had greater than 3 months of preoperative data and at least 12 months of postoperative data. Exclusion criteria included missing prescription data from the electronic medical record (EMR), primary cancer diagnosis, and procedures for infection and debridement. The study was approved by the hospital's Institutional Review Board.

Patients were categorized into two groups, preoperative opioid users (OU) and ON according to whether they had been prescribed opioids preoperatively. Opioid use was measured in morphine milligram equivalents (MME). Demographic and baseline health status were collected including age, gender, body mass index, duration of symptoms, comorbidities (including hypertension, hyperlipidemia, diabetes, coronary artery disease, depression/anxiety), length of hospital stay, duration of surgery, American Society of Anesthesiology (ASA) score, and prior surgical history. Additionally, intraoperative data including surgical invasiveness index, estimated blood loss (EBL), and duration of operation were collected.

#### Social determinants of health

Comparison between OU and ON groups were based on ten SDoH factors available in the EMR: age, gender, race, preferred language, ethnicity, work status, community median income, religious belief, insurance status, and marital status. Age was divided into quartile groups. Community median income was determined using 2019 American Community Survey data and was divided into below and above the low-income limit, as determined by the US Department of Housing and Urban Development [11,12]. The groups were also compared based on four HRB: tobacco, alcohol, and drug use, and physical activity capacity. Physical activity capacity was assessed using metabolic equivalence of task (METS) as recorded in the preoperative anesthesia visit; METS are determined based on the energy the patient is able to expend in daily tasks, such as walking 2 flights of stairs [13].

### Statistical analysis

The primary outcome measures were odds ratios for SDoH and HRB factors after univariate and multivariate logistic regression among OU versus ON patients. All statistical analyses were performed using R software version 4.1.2 with statistical significance defined at p<.05 (R Foundation, Vienna, Austria). Descriptive statistics were collected with mean and standard deviation, or median and mode where applicable. ANOVA and t-test were used to obtain p-values for all linear variables, and chi-square test was performed for categorical variables. Odds ratios were determined with a 95% confidence interval. Univariate analysis and multivariate logistic regression of SDoH and HRB were conducted against opioid use status.

## Results

Out of 521 patients that have underwent elective surgery for degenerative lumbar pathology, 16 lacked medication data at the studies designated time points and 317 patients had less than one year follow-up. A total of 188 patients were included in this cohort, with 98 included in the ON cohort and 90 in the OU cohort. The ON group had no preoperative opioid use in the 3 months prior to surgery, and the OU group had a mean preoperative opioid use of 48.2±4 MME (Table 1).

**Table 1:** Comparison of demographics between opioid naïve and opioid users. Opioid users had a higher number of prior spine surgeries, body mass index and frequency of diabetes, hypertension, coronary artery disease, and anxiety or depression compared to opioid naïve users.

Comparison of demographics between opioid naïve and opioid users				
	All	Opioid naïve	Opioid users	p-value
Age	59.7 (14.2)	59.9 (15.6)	59.4 (12.6)	.778
Number of prior spine surgeries	0.74 (1.16)	0.44 (0.84)	1.07 (1.36)	<.001
Body mass index	29.0 (5.56)	27.8 (4.90)	30.2 (6.00)	.004
Duration of symptoms (years)	6.65 (9.50)	5.59 (7.57)	7.81 (11.2)	.115
Diabetes	26 (13.8%)	8 (8.16%)	18 (20.0%)	.019
Hypertension	94 (50%)	42 (42.9%)	52 (57.8%)	.041
Hyperlipidemia	72 (38.3%)	38 (38.8%)	34 (37.8%)	.888
Coronary artery disease	10 (5.32%)	8 (8.16%)	2 (2.22%)	.070
Deep vein thrombosis	8 (4.26%)	2 (2.04%)	6 (6.67%)	.117
Anxiety/depression	73 (38.8%)	30 (30.6%)	43 (47.8%)	.016

Mean age was 59.7 years, and the cohort consisted of 46.8% women, was 82.4% white, and 11.7% Hispanic or Latinx. OU had more prior spinal surgeries (1.07 vs. 0.44, p<.001) and higher average BMI (30.2 vs. 27.8, p=.004). OU had higher levels of comorbidities, including diabetes (20% vs. 8%, p=.019), hypertension (58% vs. 43%, p=.041), and anxiety/depression (48% vs. 31%, p=.016) (Table 1). OU had higher ASA scores (2.40 vs. 2.09, p<.001) and longer length of hospital stay (3.44 days vs. 2.61 days, p=.014) (Table 2).

**Table 2:** Comparison intra and postoperative data between opioid naïve and opioid users. Opioid users had significantly higher ASA scores and longer length of hospital stay.

Comparison of surgical factors and outcomes between opioid naïve and opioid users				
	All	Opioid naïve	Opioid users	p-value
Surgical invasiveness index	6.99 (6.24)	6.33 (5.45)	7.71 (6.95)	.133
American Society of Anesthesiology Score	2.24 (0.57)	2.09 (0.50)	2.40 (0.60)	<.001
Estimated blood loss (mL)	281 (470)	233 (385)	333 (545)	.154
Total operation time (mins)	226 (133)	218 (139)	234 (125)	.416
Length of hospital stay (days)	3.01 (2.32)	2.61 (2.08)	3.44 (2.50)	.014

Univariate analysis demonstrates patients age 40 to 59 years had 3.2 times increased odds of preoperative opioid use (95% CI 1.10-10.4, p=.033). Black patients were 6.3 times more likely to use opioids before surgery (95% CI 1.01–166, p=.049). There were no increased odds based on gender or ethnicity. Those below the lower-income limit were 1.79 times more likely to have preoperative opioid use (95% CI 1.00–3.25, p=.051). Religious patients were 2.1 times more likely to use preoperatively (95% CI 1.14–3.73, p=.016). Patients on Medicaid insurance were 4.3 times more likely to have preoperative opioid use (95% CI 1.46–14.6, p=.007). There were no increased odds related to marital status (Table 3).

**Table 3:** Univariate comparison of SDoH and HRB between opioid naïve and opioid users. Univariate analysis revealed that several SDoH and HRB increased the odds of preoperative opioid use. Marital status, however, did not significantly influence the odds of preoperative opioid use.

Univariate comparison of social determinants of health and health related behaviors between opioid naïve and opioid users				
	Opioid naïve	Opioid users	Odds ratio	p-value
**Age range:**				
18–39	13 (13.3%)	6 (6.67%)	Ref.	Ref.
40–59	25 (25.5%)	38 (42.2%)	3.21 [1.10;10.4]	.033
60–75	48 (49.0%)	36 (40.0%)	1.60 [0.57;5.02]	.384
75+	12 (12.2%)	10 (11.1%)	1.77 [0.49;6.82]	.388
**Race:**				
White or Caucasian	84 (85.7%)	71 (79.8%)	Ref	Ref
Asian	6 (6.12%)	1 (1.11%)	0.22 [0.01;1.39]	.117
Black or African American	1 (1.02%)	6 (6.67%)	6.31 [1.01;166]	.049
Other	7 (7.14%)	12 (13.3%)	2.00 [0.75;5.73]	.165
**Marital status:**				
Married	71 (72.4%)	53 (58.9%)	Ref	Ref
Single	20 (20.4%)	25 (27.8%)	1.67 [0.84;3.36]	.146
Widowed	1 (1.02%)	2 (2.22%)	2.50 [0.20;80.1]	.478
Divorced	6 (6.12%)	10 (11.1%)	2.20 [0.76;6.96]	.149
**Work status:**				
Full time	44 (44.9%)	19 (21.1%)	Ref	Ref
Not employed	19 (19.4%)	45 (50.0%)	5.38 [2.55;11.8]	<.001
Retired	35 (35.7%)	26 (28.9%)	1.71 [0.82;3.64]	.156
**Median income:**				
100,000+	45 (45.9%)	25 (27.8%)	Ref.	Ref.
30,000–59,999	17 (17.3%)	22 (24.4%)	2.31 [1.04;5.23]	.04
60,000–79,999	12 (12.2%)	21 (23.3%)	3.10 [1.32;7.57]	.009
80,000–99,999	24 (24.5%)	22 (24.4%)	1.64 [0.77;3.54]	.202
**Median income dichotomous:**				
Above low-income limit	64 (65.3%)	46 (51.1%)	Ref.	Ref.
Below low-income limit	34 (34.7%)	44 (48.9%)	1.79 [1.00;3.25]	.051
**Religion:**				
Nonreligious	51 (52.0%)	31 (34.4%)	Ref.	Ref.
Religious	47 (48.0%)	59 (65.6%)	2.05 [1.14;3.73]	.016
**Insurance:**				
Private	44 (44.9%)	30 (33.3%)	Ref.	Ref.
Medicaid	5 (5.10%)	15 (17.4%)	4.26 [1.46;14.6]	.0067
Medicare	49 (50.0%)	42 (48.8%)	1.25 [0.67;2.35]	.475
Other public	0 (0.00%)	3 (3.33%)	-	-
**Smoking history:**				
Negative	62 (63.3%)	39 (43.3%)	Ref.	Ref.
Positive	36 (36.7%)	51 (56.7%)	2.24 [1.25;4.06]	.007
**METS range:**				
>5	83 (84.7%)	57 (63.3%)	Ref.	Ref.
<5	15 (15.3%)	33 (36.7%)	3.17 [1.60;6.54]	.001

Among HRB, patients who smoked were 2.2 times more likely to have preoperative opioid use (95% CI 1.25–4.06, p=.007). Those with low physical activity capacity (METS<5) were 3.2 times more likely to have preoperative opioid use (95% CI 1.60–6.54, p=.001). No association was found among alcohol use or drug use (Table 3).

Multivariate analysis demonstrated unemployment (OR 5.15, 95%CI 1.95–14.4, p<.001), lower capacity for physical activity marked by METS<5 (OR 3.76, 95%CI 1.52–9.33, p=.005), and those with community median income below the lower-income limit (OR 3.85, 95%CI 1.27–11.7, p<.001) as independent risk factors for preoperative opioid use (Table 4).

**Table 4:** Multivariate comparison of social determinants of health and health related behaviors between opioid naïve and opioid users.

	Odds ratio	p-value
**Median Income B`60,000 – 79,999**	3.85 [1.27;11.7]	.001
**Not Employed**	5.14 [1.95;14.4]	.001
**METS range <5**	3.76 [1.52;9.33]	.005

The OU cohort had a greater proportion of patients still using opioids at 3 months (83% vs. 22%, p<.001) and 1 year postoperatively (72% vs. 15%, p<.001). The OU group used more daily opioids postoperatively (57.2 MME vs. 10.0, p<.001) (Table 6). The OU group used opioids postoperatively at higher rates, at higher doses, and for a longer period of time (Figure). Analysis comparing preoperative and postoperative MME in chronic OU indicated no difference (p=.170).

**Figure 1:**
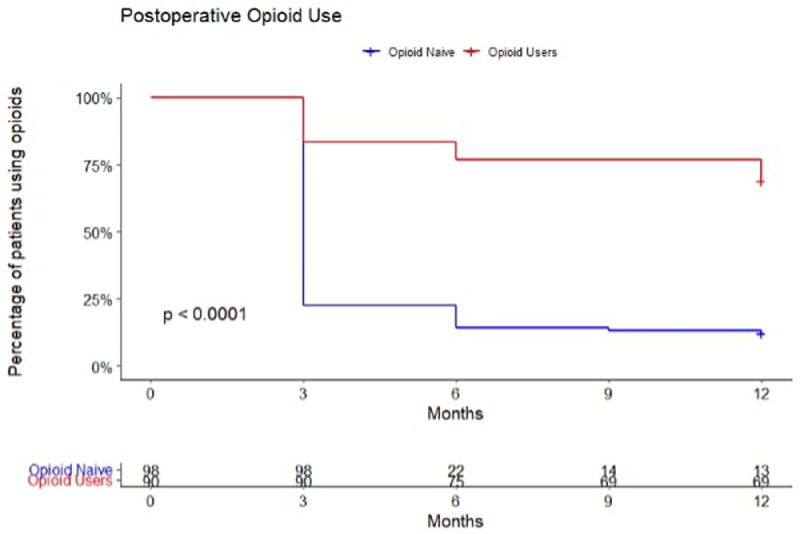
Kaplan-Meier curve of opioid use among patients undergoing spine surgery for lumbar degenerative disease.

Regarding postoperative opioid use, multivariate analysis showed that preoperative opioid use, alcohol use, and community median income below the lower-income limit were independent risk factors (p<.001, .030, and .042 respectively). Those with preoperative opioid use were 13.2 times more likely to have postoperative opioid use (95% CI 5.90–31.9, p<.001) (Table 5).

**Table 5:** Multivariate analysis of predictive factors for postoperative opioid use.

	Odds ratio	p-value
Preop opioid use	13.2 [5.90;31.9]	<.001
Alcohol use	7.03 [1.34;47.1]	.030
Below low-income limit	2.38 [1.04;5.57]	.042

**Table 6:** Comparison of postoperative opioid use between opioid naïve and opioid users. Opioid users had significantly greater opioid use at both 3 months and 1 year postoperatively compared to opioid naïve users.

	Opioid naïve	Opioid users	p-value
Preoperative morphine milligram equivalents	-	48.2 (44.8)	-
Immediately prior to surgery morphine milligram equivalents	-	48.7 (43.7)	-
Postoperative opioid use after 3 mo	22 (22.4%)	75 (83.3%)	<.001
Postoperative opioid use after 1 y	15 (15.3%)	65 (72.2%)	<.001
Postoperative morphine milligram equivalents	10.0 (27.1)	57.2 (65.7)	<.001

## Discussion

The current study evaluates preoperative opioid use among spine surgery patient who underwent elective lumbar degenerative surgery, in the context of the social factors which can affect patients’ health. Importantly, those with lower income, unemployed, or with lower physical capacity (METS<5) were more likely to use opioids preoperatively. Postoperative opioid use was strongly associated with preoperative opioid use, as well as alcohol use, and lower community median income. Furthermore, those who use opioids preoperatively used opioids postoperatively at higher rates, at higher doses, and for a longer period of time.

The opioid epidemic of the 21st century was unprecedented and had a sweeping effect on Americans, and on how doctors must handle opioids [14], [15], [16]. The effects of the opioid epidemic are especially pertinent for patients undergoing treatment for spine pathology [17], [18], [19].

Our results are consistent with previous work in this area. Previous studies have demonstrated similar associations between preoperative and postoperative opioid utilization. An analysis of the Humana database by Jain et al. observed that 87.6% of preoperative OU continued opioid use 1 year after a 1- or 2-level posterior lumbar fusion, and this has been corroborated in later studies as well [20], [21], [22]. Yerneni et al. [22] showed that outcomes were poor for those with preoperative opioid use.

Spine surgeons see a high percentage of patients who are using opioids when presenting to their clinic. Patients undergoing spine surgery have a high incidence of preoperative opioid use, ranging from 20% to 70% [23], [24], [25]. Studies continue to characterize preoperative opioid use as the single most important risk factor for long-term opioid use [20,26,27].

In order to better understand who is at risk for long-term opioid use or misuse, we must understand what risk factors contribute to their use before presentation or surgery. Studies have examined opioid use in spine clinics and patient populations but not through a social determinants lens. SDoH have shown to be reliable predictors of health outcomes in spine surgery. They have been linked to poorer outcomes in spine patients, such as postoperative complications and increased length of stay [28,29].

In this study, we have identified several social factors that are associated with preoperative opioid use. Lower physical capacity, lower community median income, and unemployment were all associated with preoperative opioid use in multivariate analysis.

Lower physical capacity, marked by METS less than 5 in patients’ preoperative anesthesia visit, was associated with preoperative opioid use with 3.8 times increased odds of opioid use. A study on stress testing indicated a METS of less than 5 as poor exercise capacity [30]. Previous studies have shown that prehabilitation can be an early intervention to decrease postoperative opioid use, and this may be an appropriate intervention for patients scored in this lower METS group [31]. Given that this risk factor is modifiable, it may be a good target for preoperative optimization in spine surgery patients. There is caution, however, of possible confounding with this metric; patients who are in more pain and therefore using more opioids may be limited in their physical capacity secondary to pain level.

Lower community median income (below the US HUD low-income limit) was also associated with preoperative opioid use, with 3.9 times increased odds. There are many historical and socioeconomic factors that can contribute to this, and the association between lower income and opioid use is well-established in the literature. Correlation has been found between low socioeconomic status and higher rates of opioid prescription renewal, higher preoperative opioid consumption in neighborhoods of lower socioeconomic status, and lower socioeconomic status and postoperative or chronic opioid use [32], [33], [34], [35]. There are hidden confounders within this variable, such as race or access to medical care [36], [37]. Access is especially difficult for subspecialty and surgical care. Additionally, when patients present at later timepoints or with more advanced disease, it is understandable that they may be in more pain or require more opioids. Late presentation is also a multifactorial problem, which is seen at higher rates among those in marginalized groups.

Similarly, in this study we found that unemployed patients had more than 5 times odds of using opioids preoperatively. Again, it is difficult to discern whether those patients are unemployed as a result of their pain, or their unemployed work status has an effect on their opioid intake [38].

Those who used opioids preoperatively had more than 13 times odds of using opioids postoperatively. This underlines the importance of the preoperative opioid use risk factors, as preoperative use has direct implications for postoperative. Additional associated risk factors for postoperative opioid use were alcohol use and community median income below the lower income limit. Lower community median income remained a risk factor from preoperative to postoperative, independent from preoperative opioid use itself. The impact of socioeconomic status on opioid use cannot be understated, and is likely multifactorial.

The main limitation of the current study is the data available, which is limited by what is collected in the EMR. As a retrospective chart review, further data that encompass other SDOH could not be obtained. Some factors which were not available include information about physical environment (transportation and housing), education (education level, language, literacy), and the patient's experience with the healthcare system. Additionally, proxies such as community median income (via zipcode) were used for socioeconomic status. Actual opioid usage was not tracked directly (such as via drug testing).

Future directions should include preoperative optimization of all factors which are modifiable. This includes prehabilitation for improvement of physical capacity, reduction of HRBs such as alcohol and smoking, and reducing preoperative opioid intake. We must also continue to focus on improving healthcare access and delivery of equitable care (care that does not vary with gender, ethnicity, or socioeconomic status); this can have an impact on patients who are unemployed, of lower socioeconomic status, or otherwise disadvantaged and presenting to spine surgeons on higher doses of opioids. The spine surgery community must continue to deliver equitable care which includes further studies of barriers to access, disparity in care, and differences in surgical outcomes. Minimizing disparity is paramount to delivering excellent, equitable care.

## Conclusions

To summarize, in adults treated with surgery for lumbar degenerative pathology, low physical activity capacity, low community median income, and unemployment were associated with preoperative opioid use. Preoperative opioid use is a strong predictor of postoperative opioid utilization at every follow-up timepoint up to 1 year after surgery. Minimizing preoperative opioid use may represent a strategy for reducing postoperative opioid use.

## Declarations of Competing Interests

The authors have no copyrighted materials included in the attached submission. The local Institutional Review Board approved this work.
